# Oppositional Cat Swarm Optimization-Based Feature Selection Approach for Credit Card Fraud Detection

**DOI:** 10.1155/2023/2693022

**Published:** 2023-01-13

**Authors:** N. Prabhakaran, R. Nedunchelian

**Affiliations:** ^1^Department of Computer Applications, Presidency College, Bangalore, India; ^2^Department of Computer Science and Engineering, Excel Engineering College, Namakkal, India

## Abstract

Credit card fraud has drastically increased in recent times due to the advancements in e-commerce systems and communication technology. Falsified credit card transactions affect the financial status of the companies as well as clients regularly and fraudsters incessantly try to develop new approaches to commit frauds. The recognition of credit card fraud is essential to sustain the trustworthiness of e-payments. Therefore, it is highly needed to design effective and accurate credit card fraud detection (CCFD) techniques. The recently developed machine learning (ML) and deep learning (DL) can be employed for CCFD because of the characteristics of building an effective model to identify fraudulent transactions. In this view, this study presents a novel oppositional cat swarm optimization-based feature selection model with a deep learning model for CCFD, called the OCSODL-CCFD technique. The major intention of the OCSODL-CCFD technique is to detect and classify fraudulent transactions using credit cards. The OCSODL-CCFD technique derives a new OCSO-based feature selection algorithm to choose an optimal subset of features. Besides, the chaotic krill herd algorithm (CKHA) with the bidirectional gated recurrent unit (BiGRU) model is applied for the classification of credit card frauds, in which the hyperparameter tuning of the BiGRU model is performed using the CKHA. To demonstrate the supreme outcomes of the OCSODL-CCFD model, a wide range of simulation analyses were carried out. The extensive comparative analysis highlighted the better outcomes of the OCSODL-CCFD model over the compared ones based on several evaluation metrics.

## 1. Introduction

With the tremendous development of e-commerce and mobile Internet techniques, online payment tools, includes credit cards have received considerable interest. While credit card brings convenience to customer, also they expose banks and cardholders to possible fraud risk [1]. Credit card fraud is a challenging issue in online payment schemes. Nilson reported that in 2023, the global fraud loss is predicted to reach $35.67 billion per annum [2]. Fraud detection and prevention are the primary means to conflict with credit card fraud. Preventing fraud contains a sequence of protocols, rules, and processes. The most widely used technique in fraud avoidance includes firewalls, secure payment gateways, and intrusion detection systems [3]. Fraud detection is carried out afterward the fraud anticipation method has been broken, which implies that fraud identification is the latter line of defence to guarantee the security of credit card transactions. Banks need to invest large amounts of money to enhance their fraud detection scheme [4] because of the necessity to defend their own business reputation and cardholders' fund. The fraud in credit card transactions arises once the stealer uses another card without the permission of corresponding individual by stealing important data such as password, PIN, and other credentials with or without the physical card. Therefore, it is a necessity for efficient and effective credit card fraud detection (CCFD) approaches to be technologically advanced that work considerably. By utilizing fraud detection modules including deep learning (DL) and machine learning, we could discover whether the forthcoming transaction is legitimate or fraud [5].

**Figure 1:**
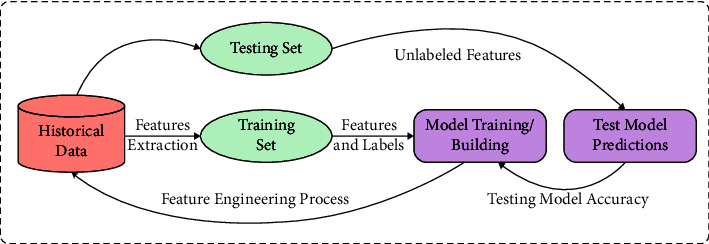
General ML process in credit fraud detection.

Machine learning (ML) and data mining (DM) are commonly utilized techniques in financial fraud detection [6]. In earlier 1998, authors start building CCFD system-based ML methods. Over several years, authors have presented various models and methods [7]. In ML method, CCFD is a standard binary classification problem. The detection scheme is intended at defining whether the present transaction is fraudulent or legal according to the past transaction history [8]. Different approaches were introduced for tackling these problems; including semisupervised, supervised, and unsupervised learning. ML is an algorithm that handles the technique that provides the computer, the ability to advance and study from the experience without being explicitly programmed. The general process involved in the ML-based CCFD is shown in Figure 1. ML is the most used and treading technology due to its less time consumption, different applications, and precise results [9].

As an example, consider diagnosis, regression, medical, and so on. ML includes the integration of statistical models and an algorithm that allows computer to execute the operation without hard coding, then it is tested on the trained model, and then a model is built through training data. The DL is a branch of the ML technique that employs a neural network (NN) system. A few technologies that belong to DL methods include recurrent neural network (RNN), convolutional neural networks (CNN), artificial neural networks (ANN), autoencoder, and so on [10]. DL uses an NN system that resembles the human brain in making decisions and processing the data. All metaheuristic methods find a balance between local and global searches (intensification and randomization) to some degree. These flexible metaheuristic methods are based on nature and are used to solve high-dimensional, nonlinear optimization problems like task-resource assignment. With these methods, all the information about the population can be used to find specific solutions. So far, a lot of attention has been paid to the theory of evolution.

In-depth analysis and a performance assessment of the cat swarm optimization CCSO algorithm are presented in this paper. Since its introduction, OCSO has received a great deal of praise for being a reliable and effective metaheuristic swarm-based optimization approach. It has been used to tackle numerous optimization issues, and numerous variations of it have been developed. There is not a thorough analysis or performance evaluation of this in the literature, though. To review all these works, including their developments and applications, this paper has grouped them into various categories. Additionally, OCSO is examined using 10 contemporary benchmark functions and 23 benchmark functions from the past (CEC 2019). The outcomes are then contrasted with three cutting-edge and potent optimization algorithms: fitness dependent-optimizer (FDO), the butterfly optimization algorithm (BOA), and the dragonfly algorithm (DA) (FDO). The algorithms are then ranked using the Friedman test, and the findings indicate that OCSO comes out on top overall. Finally, statistical methods are used to support the superior performance of the OCSO algorithm.

The motivation for this research stems from the fact that the performance of various machine learning classifiers for the credit card fraud detection challenge has not been thoroughly investigated in the past. Furthermore, it was discovered that the performance of nature-inspired metaheuristics can be investigated further for ML tuning and training. As a result, in addition to the proposed approach, other recent state-of-the-art OCSODL-CCFD techniques have been implemented and adapted, and their performance in tuning three ML models for the practical and important credit card fraud detection problem has been thoroughly examined. As a result, this manuscript includes a comprehensive comparison of three ML methods and several metaheuristics. Based on the foregoing, the basic research question that guided the experimentation presented in this paper is whether it is possible to improve the detection of malicious credit card activities by using ML models and to improve the classification performance of SVM, ELM, and XGBoost methods by tuning them with the OCSODL-CCFD technique.

This study presents a novel oppositional cat swarm optimization-based feature selection with the deep learning model for CCFD, called the OCSODL-CCFD technique to detect and classify fraudulent transactions using credit cards. The main contributions of the proposed research can be summarized as follows: The creation of a novel, improved version of the well-known OCSODL-CCFD technique that addresses the original implementation's known flaws. The use of the developed algorithm to tune three machine learning classifiers for the specific task of fraud detection with the goal of improving the classifiers' accuracy as well as other performance metrics. A thorough comparison of various swarm intelligence metaheuristics for ML tuning against a real-world credit card fraud challenge. The OCSODL-CCFD technique primarily designs a feature selection technique using the OCSO algorithm. In addition, the chaotic krill herd algorithm (CKHA) with a bidirectional gated recurrent unit (BiGRU) model is utilized for classification purposes. The design of OCSO and CKHA algorithms aid in minimizing the computational complexity and enhancing the classification performance. To demonstrate the better efficiency of the OCSODL-CCFD technique, an experimental result analysis is made on a benchmark dataset.

## 2. Related Works

Xie et al. [11] presented a heterogeneous ensemble learning method that enabled data distribution (HELMDD) to handle class imbalance issues in CCFD. They authenticate the efficacy of HELMDD on 2 real-time credit card data sets. The experiment result demonstrates that HELMDD technique has obtained effective outcomes over the existing methods. Asha and Kumar [12] intended at utilizing the various approaches of ML include ANN, support vector machine (SVM), and *k* nearest neighbor (KNN) in forecasting the incidence of the fraud. Furthermore, they conducted a distinction of the accomplished supervised ML and DL approaches for differentiating among fraud and nonfraud transactions. ML algorithm is utilized for detecting credit card frauds. First, the typical model is employed. Next, a hybrid method that uses AdaBoost and the majority voting method is employed. In order to estimate the efficacy of the system, an open-source credit card dataset is applied. Next, a real-time credit card dataset is examined. Additionally, noise is included in the data sample for additionally assessing the strength of the algorithm.

In [13], an outlier detection method is presented to solve the problem by utilizing supervised and unsupervised ML approaches. The efficiency of four distinct approaches is evaluated by attaining scores of assessment metrics. Handa et al. [14] introduced a hybrid analysis of distinct ML algorithms in detecting fraud transactions. Then, discuss and compare the performances of DL, supervised, unsupervised, and hybrid methods executed by ensemble ML methods. The original data set attained from the online community is balanced by utilizing sampling technique. The hybrid analysis result shows that the supervised ensemble models perform effectively when compared to the other algorithms. Lenka et al. [15] designed a fraud detection scheme with an ensemble approach. In the suggested method, the imbalanced credit card data set is initially balanced by the random undersampling method, next the efficiency of the system is estimated by the ensemble and single-base classifiers.

Hussein et al. [16] proposed the integration of different classifications via a stacking ensemble model for detecting credit card fraud. The sequential minimal optimization and fuzzy-rough nearest neighbor are used as base classifiers. The collective predictions become data input for the metaclassifiers, which is logistic regression resultant in the last prediction result for enhanced detection. The experiment results undergone comparison with 7 approaches affirms that the ensemble method could effectively identify credit card fraud. The researchers in [17] presented an ensemble method-based sequential modeling of data using DRNN and a novel voting method-based ANN to identify fraudulent action. Additionally, we presented a novel approach to train the above-mentioned voting mechanism. Preitl and Precup [18] discusses some of the most important aspects of multiparametric quadratic programming (mp-QP) problems. Model Predictive Control (MPC) is a specific mp-QP problem, and this powerful tool is used for control and simulation in a case study. Because mp-QP solutions can be expressed as piecewise affine linear functions of the state, a new implementation in the form of adaptive network-based fuzzy inference systems is proposed. The presentation focuses on the double integrator plant, which appears frequently in case studies (electrohydraulic servosystem). Zamfirache et al. [19] proposed a novel Policy Iteration Reinforcement Learning (PI RL-based control approach that trains the policy NN using a metaheuristic GWO algorithm. The new GWO-approach was validated on a nonlinear servo system position control experimental platform against two other approaches that used the GD and PSO algorithms, respectively.

Aricán and Aydin [20] there are numerous studies that are available in the literature on the topic of object detection, which is a very hot topic in computer vision. The community now has easy access to 3D data thanks to technological and scientific advances, making 3D descriptor an important subject. In this study, a new 3D descriptor is produced by the system by fusing depth data from RGB-D and BoVW. This method does away with the drawbacks of BoVW, and tests demonstrate that it provides a higher accuracy rate than the original BoVW method. As a result, the proposed 3D descriptor performs well when used with 3D datasets like those from the Kinect for 3D object detection. Borlea et al. [21] this paper presents a way of improving the resulted clusters generated by the *K*-means algorithm by postprocessing the resulted clusters with a supervised learning algorithm. The proposed approach is focused on improving the quality of the resulting clusters and not on reducing the processing time.

## 3. Materials and Methods

### 3.1. The Proposed Model

**Figure 2:**
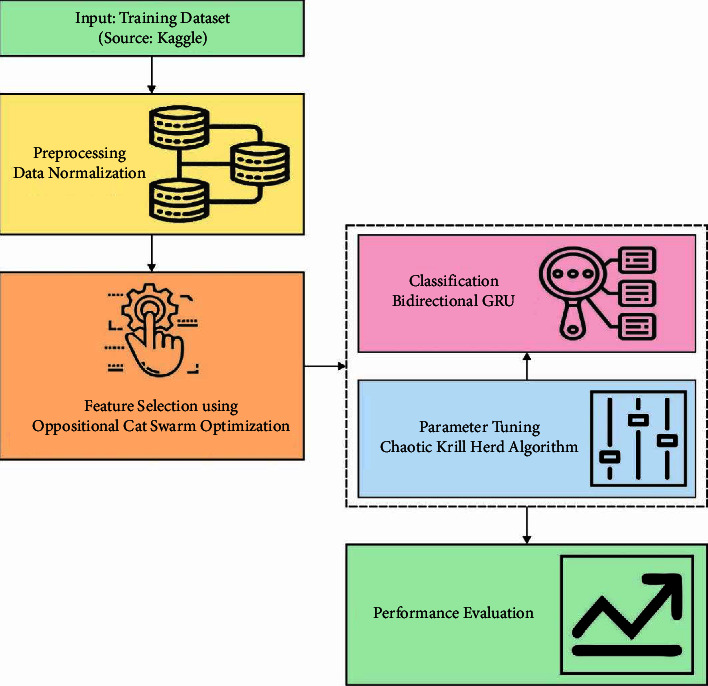
Working principle of OCSODL-CCFD technique.

In this study, a novel OCSODL-CCFD technique is designed to identify and classify fraudulent transactions using credit cards. The working principle of OCSODL-CCFD technique is shown in Figure 2. The proposed OCSODL-CCFD technique encompasses different subprocesses namely preprocessing, OCSO-based election of features, BiGRU classifier, and CKHA-based hyperparameter optimizer.

### 3.2. Preprocessing

In any data classification problem, the quality of the input data plays a major role, which necessitates the preprocessing step. Data normalization is a commonly employed process to preprocess the input data. Primarily, data is preprocessed by the use of min-max normalization approach, which rescaled the input values into a range of values, i.e. [0, 1] or [−1, 1]. It can be presented as follows:

(1) $$\begin{array}{c} y^{\prime }=\left(y_{\max}-y_{\min}\right)\times \frac{\left(x_{i}-x_{\min}\right)}{\left(x_{\max}-x_{\min}\right)}+x_{\min}\text{.} \end{array}$$

In which (*y*_max_ − *y*_min_)=0; when (*x*_max_ − *x*_min_)=0.

### 3.3. Algorithmic Process of OCSO-FS Technique

The OCSO demonstrated its aptitude for handling various, difficult issues in various contexts. But the OCSO algorithm has advantages and disadvantages, just like any other metaheuristic algorithm. While the seeking mode resembles a local search, the tracing mode resembles a global search. This algorithm benefits greatly from the separation and independence of these two modes. This makes it possible for researchers to quickly alter or enhance these modes and thus achieve a proper balance between the phases of exploration and exploitation. This algorithm's quick convergence is another benefit, which makes it a good choice for applications that demand prompt responses. However, the algorithm has a high likelihood of entering local optima, also known as premature convergence, which can be thought of as the algorithm's primary flaw. During the feature selection process, the preprocessed credit card data are passed into the OCSO-FS technique to choose an optimal feature subset. The CSO algorithm is an optimization approach in the SI [18]. The CSO approach model the behaviors of cat into two modes: “Tracing mode” and “Seeking mode.” In CSO, we utilize cats as particles to resolve the problem. In CSO, all the cats have their own location made up of *D* dimension, velocity for all the dimensions, fitness values that denotes accommodating cats to the FF, and a flag to recognize whether the cat is in tracing or seeking modes. The last solution will be the optimal location of a cat. The CSO keeps the optimal solutions till it reaches the ending condition [18]. To model the cat's behavior in resting time and being alert, we utilize this model. It is a time for deciding and thinking about further steps. The procedure of seeking mode is described in the following:

Step l: Make *j* copy of the existing location of cat_*k*_, whereas *j*=SMP. When the values of SPC are true, consider *j*=(SMP−1), which retains the existing location as one of the candidates.
Step 2: For all the copies, as per CDC, random plus or minus SRD percent the existing values and replace the old one.
  (2) $$\begin{array}{c} Xjd_{new}=\left(1+rand*SRD\right)*Xjd_{old}\text{,} \end{array}$$
where Xjd_old_ is the current position; Xjd_new_ is the next position; *j* denotes the number of a cat and *d* denotes the dimensions; and rand is a random number in the interval of [0, 1].
Step 3: Evaluate the fitness value (FS) of each candidate point.
Step 4: When each FS is not accurately equivalent, evaluate the selected possibility of candidate point using equation (3), or else set the selected possibility of candidate point to be 1.
Step 5: Arbitrarily elect the point for moving from candidate point, and replaces the location of cat_*k*_.
  (3) $$\begin{array}{c} P_{i}=\frac{\left|SSE_{i}-SSE_{\;\max\;}\right|}{SSE_{\;\max\;}-SSE_{\;\min\;}}\text{.} \end{array}$$

While the aim of the FF is to determine the minimal solution, *FS*_*b*_=*FS*_ max  _, or else *FS*_*b*_=*FS*_min_. In the tracing mode, cats desired to trace foods and targets. The procedure can be mentioned in the following:

Step 1: Upgrade the velocity for all the dimensions as per equation (4).
Step 2: Verify whether the velocity within the interval of maximal velocity. If novel velocity is in over range, it is fixed equivalent to limits.
  (4) $$\begin{array}{c} V_{k,d}=V_{k,d}+r_{1}c_{1}\left(X_{best,d}-X_{k,d}\right)\text{.} \end{array}$$
Step 3: upgrade the location of cat_*k*_ as per the following equation:
  (5) $$\begin{array}{c} x_{k,d}=x_{k,d}+V_{k,d}\text{.} \end{array}$$
*X*_bestd_ represent the location of the cat, which has the optimal fitness values, *X*_*k*,*d*_ represent the location of cat_*k*_,*c*_1_ indicates an acceleration coefficient to extend the cat velocity to move in the solution space and is corresponding to 2.05 and *r*_1_ represent an arbitrary value within [0, 1].

In order to enhance the outcomes of the CSO algorithm, the OCSO algorithm has been derived based on the population initialization using oppositional-based learning concepts [22]. The mathematical process of OCSO-FS technique was established. Usually, the classifier (for instance, supervised learning) of some data sets that have size *N*_*S*_ × *N*_*F*_ where *N*_*S*_ implies the amount of samples and *N*_*F*_ signifies the amount of features. An important function of FS problem is for selecting a subset of features *S* in entire amount of features (*N*_*F*_) whereas the size of *S* is lesser than *N*_*F*_. It could be attained with minimized the subsequent main function:

(6) $$\begin{array}{c} Fit=\lambda \times \gamma_{S}+\left(1-\lambda \right)\times \left(\frac{\left|S\right|}{N_{F}}\right)\text{,} \end{array}$$

where *γ*_*S*_ defines the classifier error utilizing *S* and |*S*| are the amount of chosen features. *λ* demonstrates the utilized for balancing among (|*S*|/*N*_*P*_) and *γ*_*S*_.

### 3.4. BiGRU-Based Credit Card Fraud Classification

After the selection of features, they are fed into the BiGRU model to detect and classify credit card frauds. Due to the difficult infrastructure of long short term memory (LSTM) units, there is a challenge of long training time [23, 24]. The GRU memory unit integrates the forgetting gate *f* and input gate *i* from the LSTM to the update gate *z* that not only recollects essential features, among them, resolve the long dependence issue, but the infrastructure was easy as LSTM. At time *n*, to provide input *X*_*n*_, the hidden layer of GRU output *h*_*n*_, the particular computation procedure is as follows:

(7) $$\begin{array}{c} z_{n}=\sigma \left(W_{Z}\cdot \left[h_{n-1},x_{n}\right]\right)\text{,}\\ r_{n}=\sigma \left(W_{r}\cdot \left[h_{n-1},x_{n}\right]\right)\text{,}\\ {\tilde{h}}_{n}=\tanh\left(W\cdot \left[r_{n}*h_{n-1},x_{n}\right]\right)\text{,}\\ h_{n}=\left(1-z_{n}\right)*h_{n-1}+z_{n}*{\tilde{h}}_{n}\text{,} \end{array}$$

where *W* implies the weight matrix linking the 2 layers, *σ* and tan *h* refer the activation function. *z* and *r* stand on the update and reset gates correspondingly. In order problem, the typical RNN utilizes the preceding data based on the forward input order, however doesn't consider the following data. Following this issue, the BiRNN technique presented [25] whereas memorized the above data, also memorizing the subsequent data. The fundamental purpose is for utilizing 2 RNN for processing the forward as well as reverse sequences correspondingly. The output is then linked to similar resultant layer and bidirectional context data to the feature sequence was recorded. According to the BiRNN, the BiGRU technique was achieved by exchanging the hidden layer neuron from BiRNN with GRU memory units. To provide *n*_*o*_ dimension input (*x*_1_, *x*_2_,…, *x*_*n*_*o*__). At time *n*, the hidden layer of BGRU output *h*_*n*_. The computation procedure is as follows:

(8) $$\begin{array}{c} \overset{⟶}{h_{n}}=\sigma \left(W_{x\overset{⟶}{h}}x_{n}+W_{\overset{⟶}{h}\overset{⟶}{h}}\overset{⟶}{h_{n-1}}+b_{\overset{⟶}{h}}\right)\text{,}\\ \overset{´}{h_{n}}=\sigma \left(W_{x\overset{⃖}{h}}x_{n}+W_{\overset{⃖}{h}\overset{⃖}{h}}\overset{´}{h_{n-1}}+b_{\overset{⃖}{h}}\right)\text{,}\\ h_{n}=\overset{⟶}{h_{n}}⊕\overset{´}{h_{n}}\text{,} \end{array}$$

where *W* implies the weight matrix linking the 2 layers, *b* stands for the bias vectors, *σ* represents the activation functions, $\overset{⟶}{h_{n}}$ and $\overset{⃖}{h_{n}}$ refers the output of positive as well as negative GRU correspondingly. ⊕ signifies the element‐wise sum.

### 3.5. CKHA-Based Hyperparameter Tuning

Extensive research has been conducted to determine the mechanisms that cause marine animal populations to form nonrandom patterns. Several mechanisms have been identified, including predator protection, feeding, environmental characteristics, and improved reproduction. The Antarctic krill is one of the most studied marine species. In fact, there are several uncertainties about the krill herd's representative distribution. Several conceptual frameworks have been proposed to explain the krill herd pattern. The findings indicate that krill swarms are the primary organisational unit of this species.

Individual krill are attacked by marine predators such as sea birds and penguins by leading them to areas with lower krill density. Following a predatory attack, krill herd formation has two primary goals: (1) increase krill density and (2) increase access to food. The objective function has been identified as krill behaviour to increase density and locate food. Herding is then observed around local minima. Individual krill movement is such that the best solution in this search for food and increased density can be found.

To effectually adjust the hyperparameters involved in the CKHA technique, an effective hyperparameter tuning process takes place using the CKHA. KH is a metaheuristic optimized technique employed for resolving optimized problems which are according to stimulation of the herd of krill swarms regarded environmental and biological processes [26, 27]. The time‐based place of separate krill from 2D surface is provided as follows:

1. Motion induced by krill individual;
2. Foraging effort
3. Physical or arbitrary diffusion

The Lagrangian process was generalization to *n* dimensional decision region.

(9) $$\begin{array}{c} \frac{dX_{i}}{dt}=N_{i}+F_{i}+D_{i}\text{,} \end{array}$$

where as *N*_*i*_ refers the movement induced by krill individual; *F*_*i*_ signifies the foraging process; and *D*_*i*_ represents the physical diffusion of *i*^th^ krill individuals. The motion induced by another krill individual's, the way of induced process, *α*_*i*_ has been estimated by local swarm density (local effect), repulsive swarm density (repulsive effect), and targeted swarm density (targeted effect).

(10) $$\begin{array}{c} N_{i}^{new}=N^{ma\times }\alpha_{i}+\omega_{n}N_{i}^{old}\text{.} \end{array}$$

Assume that *N*^maks^ be the higher induced speeds, *N*^old^ signifies the last induced process, *ω*_*n*_ represents the inertia weight of induced process is zero and one. The foraging process was determined as 2 important factors. The food place and preceding experience regarded the food place:

(11) $$\begin{array}{c} F_{i}=V_{f}\beta_{j}+\omega_{f}F_{i}^{old}\text{,} \end{array}$$

where as

(12) $$\begin{array}{c} \beta_{j}=\beta_{j}^{food}+\beta_{j}^{best}\text{.} \end{array}$$

*ω*_*f*_ implies the inertia weight of foraging process amongst zero and one, *F*_*i*_^old^ refers the last foraging process and *V*_*f*_ stands for the foraging speed. The physical diffusions of krill individuals were managed as an arbitrary technique. This effort was defined as dependent upon arbitrary directional vector and higher diffusion speeds.

(13) $$\begin{array}{c} D_{i}=D^{ma\times }\delta \text{.} \end{array}$$

While *δ* denotes the arbitrary directional vectors, and *D*^ma×^ implies the maximal diffusion speed and the range of one and one. By the above-mentioned motion, effectual parameter of motion, the location vector of krill individual at time *t* to *t*+△*t* are formulated as

(14) $$\begin{array}{c} X_{i}\left(t+△t\right)=X_{i}\left(t\right)+△t\frac{dX_{i}}{dt}\text{.} \end{array}$$

Mention that △*t* is most essential constant and is wisely regulated with respect to providing practical optimization issues. This parameter is assumed the scale factor of speed vectors. △*t* completely depend on the search space and it appears that simply achieved in the subsequent written as

(15) $$\begin{array}{c} △t=c_{t}\overset{NV}{\underset{j=1}{\sum }}\left(UB_{j}-LB_{j}\right)\text{,} \end{array}$$

where *NV* refers the entire amount of variables and *UB*_*j*_ and *LB*_*j*_ implies the upper as well as lower bounds of *j*^th^ variable (*j* = 1,2, . . . , *NV*) correspondingly. Therefore, the absolute of its subtraction illustrates the search spaces as shown in Algorithm 1.

Very specific, low value of *C*_*ζ*_ create the krill individuals carry out the search from the space carefully. In random-based optimized techniques, the techniques utilizing chaotic variables rather than arbitrary variables are named the chaotic optimization algorithm (COA). During these techniques, as chaos is the feature of nonrepetition and ergodicity, it is implemented entire searches at maximum speed than stochastic search which depend on probability. For accomplishing this matter, here 1D noninvertible map is utilized for producing chaotic set. During the current analysis, the subsequent 13 well‐known 1D chaotic maps are executed for generating CKHA. The CKHA approach resolves a fitness function for reaching increased classifier efficacy. It defines a positive integer for representing an optimum execution of the candidate solution. In this case study, the minimized classification error rate was regarded as fitness is provided in equation (16). A better solution is a minimal error rate and the worst solution gains a superior error rate.

(16) $$\begin{array}{c} fitness\left(x_{i}\right)=Classifier\;Error\;Rate\left(x_{i}\right)=\frac{number\;of\;misclassified\;instances}{Total\;number\;of\;instances}*100\text{.} \end{array}$$

## 4. Results and Discussion

**Figure 3:**
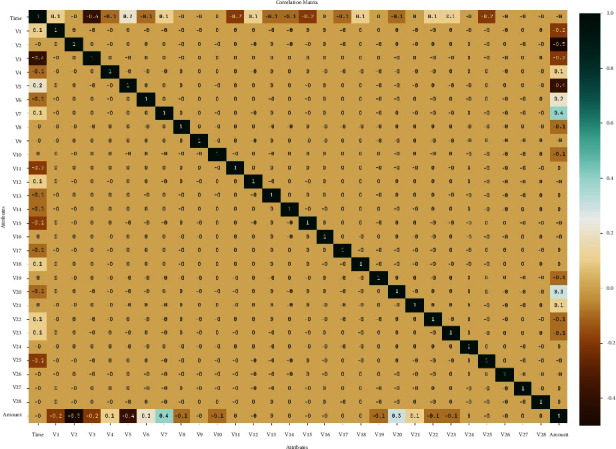
Correlation matrix.

This section investigates the CCFD result analysis of the OCSODL-CCFD technique using benchmark dataset from the Kaggle repository [28, 29]. It holds a set of 284807 transactions, comprising two classes, namely, fraud and nonfraud. The correlation matrix of the applied dataset is shown in Figure 3. The credit card fraud detection dataset, which can be downloaded from Kaggle, was used in this study. This dataset includes two-day transactions made in September 2013 by cardholders in Europe. There are 31 numerical features in the dataset. Given that some of the input variables contain financial data, the PCA transformation of these input variables was carried out to maintain the anonymity of the data. The given features were not transformed for three of them. The “Time” feature displays the elapsed time between the dataset's first transaction and each subsequent transaction. The feature “Amount” refers to the total amount of credit card transactions. In this study, we make use of a dataset that records credit card purchases made by European cardholders over the course of two days in September 2013. In total, there are 284807 transactions in this dataset, and 0.172% of them are fraudulent. The dataset contains the 30 features Time and Amount (V1,…, V28). The dataset's attributes are all numerical in nature. The class (type of transaction) is represented by the final column, where a value of 1 indicates a fraudulent transaction and a value of 0 otherwise. For data security and integrity reasons, the features V1 to V28 are not named. We used the Synthetic Minority Oversampling Technique (SMOTE) method in the data preprocessing stage of the suggested framework to address the problem of class imbalance. By choosing samples that are close to one another in the feature space, the SMOTE method creates a new instance of the minority class at a point along the line and draws a line between the data points.

**Figure 4:**
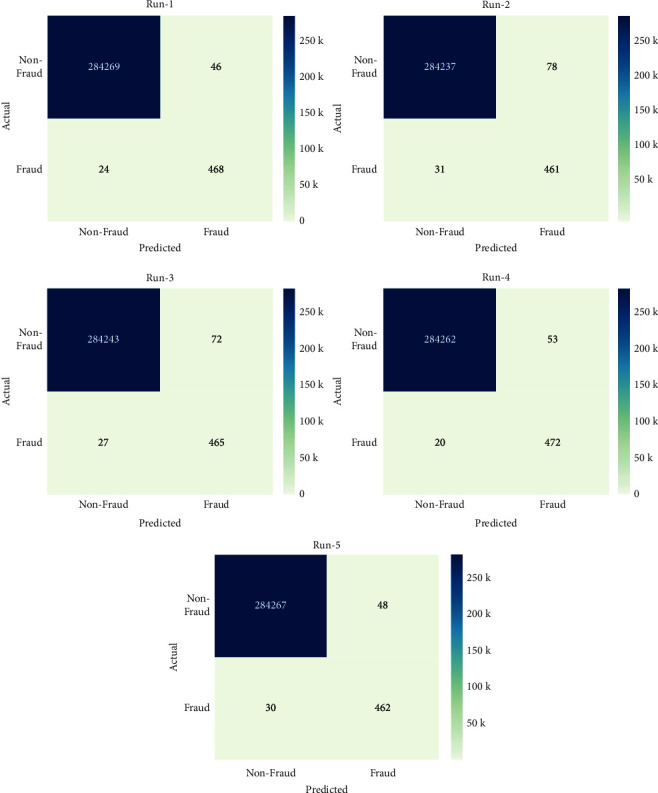
Confusion matrices of OCSODL-CCFD technique.

Figure 4 exhibited the confusion matrix generated by the OCSODL-CCFD method under distinct runs. The figure indicated that the OCSODL-CCFD method has effectually recognized the samples into nonfraud and fraud classes. For example, under run-1, the OCSODL-CCFD approach has categorized 284269 samples into non-fraud and 468 samples into fraud. Additionally, with run-3, the OCSODL-CCFD technique has recognized 284243 samples into nonfraud and 465 instances into fraud. Lastly, with run-1, the OCSODL-CCFD technique has categorized 284267 samples into nonfraud and 462 instances into fraud.

**Figure 5:**
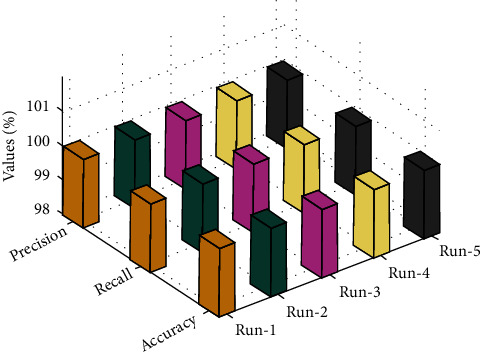
Pre*c*_*n*_, rec*a*_*l*_, and acc*u*_*y*_ analysis of the OCSODL-CCFD technique.

Table 1 provides an overall CMFD result assessment of the OCSODL-CCFD method with several runs.  Figure 5 investigates the pre*c*_*n*_, rec*a*_*l*_, and acc*u*_*y*_ examination of the OCSODL-CCFD model with different runs. The results indicated that the OCSODL-CCFD technique has obtained effectual outcomes under every run. For example, on run-1, the OCSODL-CCFD model has resulted to pre*c*_*n*_, rec*a*_*l*_, and acc*u*_*y*_ of 99.99%, 99.98%, and 99.98%, respectively. Concurrently, with run-3, the OCSODL-CCFD technique has accomplished pre*c*_*n*_, rec*a*_*l*_, and acc*u*_*y*_ of 99.99%, 99.97%, and 99.97%, respectively. Simultaneously, with run-5, the OCSODL-CCFD technique has attained pre*c*_*n*_, rec*a*_*l*_, and acc*u*_*y*_ of 99.99%, 99.98%, and 99.97%, respectively.

**Figure 6:**
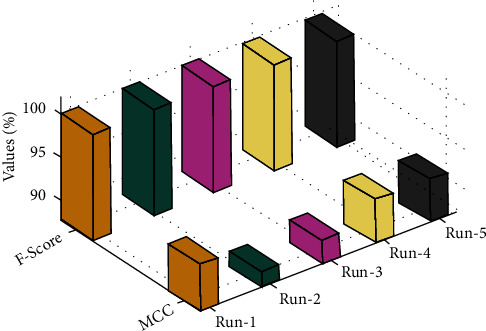
*F*_score_ and MCC analysis of the OCSODL-CCFD technique.

Figure 6 demonstrates *F*_score_ and MCC inspection of the OCSODL-CCFD model on different runs. The result portrayed that the OCSODL-CCFD technique has extended better results under every run. For example, on run-1, the OCSODL-CCFD technique has reached to *F*_score_ and MCC of 99.99%, and 93.05%, respectively. At the same time, with run-3, the OCSODL-CCFD technique has offered *F*_score_ and MCC of 99.98% and 90.45%, respectively. Also, with run-5, the OCSODL-CCFD technique has provided *F*_score_ and MCC of 99.99% and 92.22% respectively.

**Figure 7:**
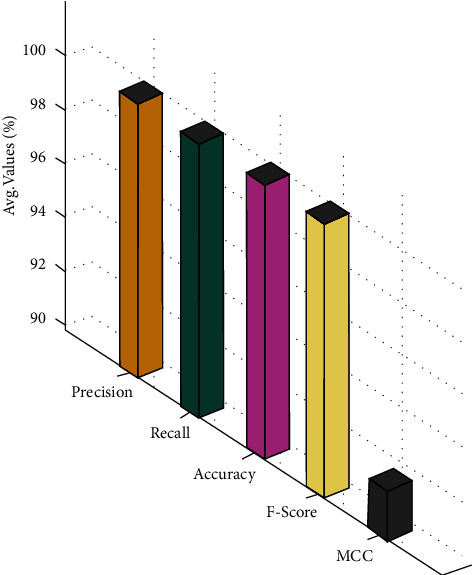
Average CCFD result analysis of the OCSODL-CCFD technique.

Figure 7 illustrates the average CCFD result examination of the OCSODL-CCFD method. The figure shows that the OCSODL-CCFD method has reached improved average classifier results with the average pre*c*_*n*_, rec*a*_*l*_, acc*u*_*y*,_*F*_score_, and MCC of 99.99%, 99.98%, 99.97%, 99.99%, and 91.62%, respectively.

**Figure 8:**
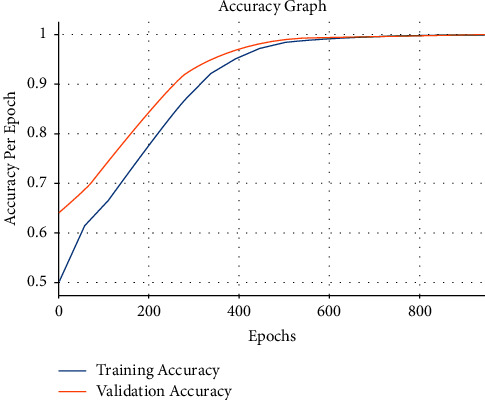
Accuracy analysis of OCSODL-CCFD technique.

Figure 8 demonstrates the accuracy inspection of the OCSODL-CCFD method on the test dataset applied. The figure portrayed that the OCSODL-CCFD technique has depicted enhanced training and validation accuracies.

**Figure 9:**
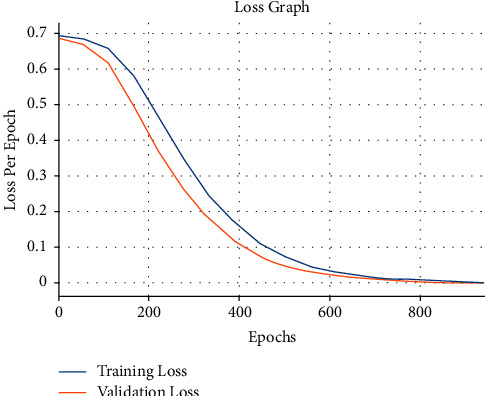
Loss analysis of OCSODL-CCFD technique.

A brief loss graph examination of the OCSODL-CCFD technique on the test dataset is reported in Figure 9. From the results, it is observable that the OCSODL-CCFD technique has gained minimal training and validation loss.

For ensuring the enhanced outcomes of the OCSODL-CCFD method, a comparative analysis [2] is made in Table 2.

**Figure 10:**
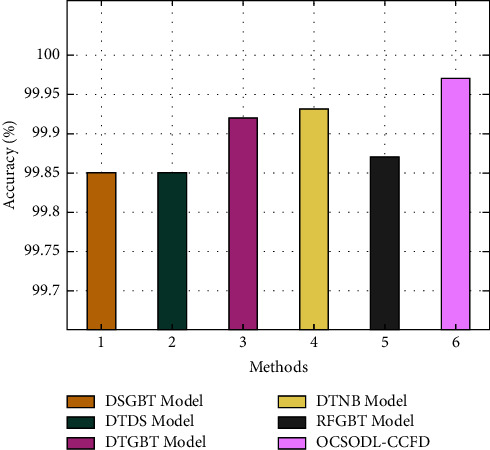
Comparative accuracy analysis of OCSODL-CCFD technique.

Figure 10 showcases the accuracy analysis of the OCSODL-CCFD technique with other methods. The experimental values show that the DSGBT and DTDS techniques have obtained slightly reduced accuracy of 99.85% and 99.85%. In line with, the RFGBT technique has resulted in moderately improved accuracy of 99.87%. Though the DTNB and RFGBT techniques have accomplished near optimal accuracy of 99.93% and 99.92%, the proposed OCSODL-CCFD technique has depicted higher accuracy of 99.97%.

**Figure 11:**
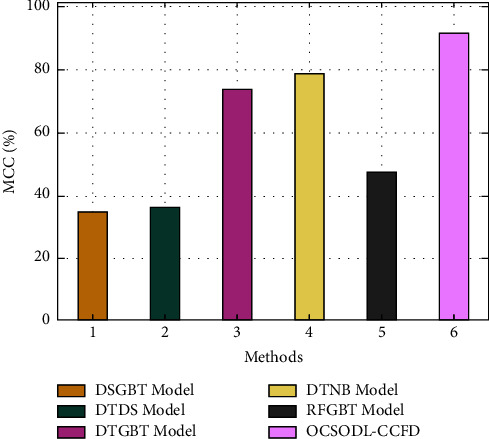
Comparative MCC analysis of OCSODL-CCFD technique.

Figure 11 exhibits the MCC analysis of the OCSODL-CCFD technique with recent methods. The figure reported that the DSGBT and DTDS techniques have resulted in certainly minimal MCC of 34.30% and 36.10%. Along with that, the RFGBT technique has attained somewhat enhanced MCC of 46.80%. Though the DTNB and RFGBT techniques have reached competitive MCC values of 78.80% and 73.70%, the proposed OCSODL-CCFD technique has demonstrated maximum of 91.62%. From these results and discussion, it is assumed that the OCSODL-CCFD method has appeared as an effective tool for CCFD.

## 5. Conclusions

In this article, a novel OCSODL-CCFD technique has been designed to detect and classify fraudulent transactions using credit cards. The proposed OCSODL-CCFD technique encompasses different subprocesses, namely, preprocessing, OCSO-based election of features, the BiGRU classifier, and the CKHA-based hyperparameter optimizer. The design of the OCSO algorithm helps to reduce the computational complexity and boost the classification results. Besides, the CKHA assists in optimally choosing the hyperparameter values of the BiGRU model. For showcasing the better efficiency of the OCSODL-CCFD technique, an experimental result analysis is made on benchmark dataset. A wide-ranging comparison study reported the better outcomes of the OCSODL-CCFD technique over the compared methods in terms of different measures. The experimental results that were achieved using the OCSO-selected attributes demonstrated that the OCSODL-CCFD techniques achieved an overall optimal accuracy of 99.97%. In the future, data clustering and outlier detection approaches can be designed to boost the classifier results.

## Figures and Tables

**Algorithm 1:**
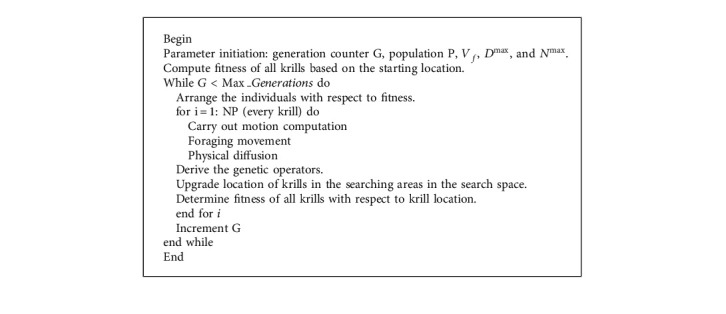
Pseudocode of KHA.

**Table 1:** Overall CMFD results of OCSODL-CCFD technique.

Runs	Precision	Recall	Accuracy	*F*-score	MCC
Run-1	99.99	99.98	99.98	99.99	93.05
Run-2	99.99	99.97	99.96	99.98	89.50
Run-3	99.99	99.97	99.97	99.98	90.45
Run-4	99.99	99.98	99.97	99.99	92.86
Run-5	99.99	99.98	99.97	99.99	92.22
Average	99.99	99.98	99.97	99.99	91.62

**Table 2:** Comparative CCFD results analysis of OCSODL-CCFD technique.

Methods	Accuracy	MCC
DSGBT model	99.85	34.30
DTDS model	99.85	36.10
DTGBT model	99.92	73.70
DTNB model	99.93	78.80
RFGBT model	99.87	46.80
OCSODL-CCFD	99.97	91.62

## Data Availability

The data that support the findings of this study are available from the corresponding author upon reasonable request.
